# BK channel agonist represents a potential therapeutic approach for lysosomal storage diseases

**DOI:** 10.1038/srep33684

**Published:** 2016-09-27

**Authors:** Xi Zoë Zhong, Xue Sun, Qi Cao, Gaofeng Dong, Raphael Schiffmann, Xian-Ping Dong

**Affiliations:** 1 Department of Physiology and Biophysics, Dalhousie University, Sir Charles Tupper Medical Building, 5850 College Street, Halifax, B3H 4R2, Nova Scotia, Canada; 2 Institute of Metabolic Disease, Baylor Research Institute, 3812 Elm Street, Dallas, TX, 75226, USA

## Abstract

Efficient lysosomal Ca^2+^ release plays an essential role in lysosomal trafficking. We have recently shown that lysosomal big conductance Ca^2+^-activated potassium (BK) channel forms a physical and functional coupling with the lysosomal Ca^2+^ release channel Transient Receptor Potential Mucolipin-1 (TRPML1). BK and TRPML1 forms a positive feedback loop to facilitate lysosomal Ca^2+^ release and subsequent lysosome membrane trafficking. However, it is unclear whether the positive feedback mechanism is common for other lysosomal storage diseases (LSDs) and whether BK channel agonists rescue abnormal lysosomal storage in LSDs. In this study, we assessed the effect of BK agonist, NS1619 and NS11021 in a number of LSDs including NPC1, mild cases of mucolipidosis type IV (ML4) (TRPML1-F408∆), Niemann-Pick type A (NPA) and Fabry disease. We found that TRPML1-mediated Ca^2+^ release was compromised in these LSDs. BK activation corrected the impaired Ca^2+^ release in these LSDs and successfully rescued the abnormal lysosomal storage of these diseases by promoting TRPML1-mediated lysosomal exocytosis. Our study suggests that BK channel activation stimulates the TRPML1-BK positive reinforcing loop to correct abnormal lysosomal storage in LSDs. Drugs targeting BK channel represent a potential therapeutic approach for LSDs.

Lysosomes are the cell’s recycling centers in which lysosomal enzymes digest endocytosed or excess materials. Emerging evidence suggests that lysosomal membrane proteins including ion channels and transporters also play essential roles in material recycling by creating optimal environment for the enzymes or regulating lysosome membrane trafficking. Deficiencies in either the enzymes or membrane proteins results in a group of inherited metabolic disorders known as the lysosomal storage disorders (LSDs)^1^,^2^. Although enzyme replacement is regarded as a promising therapeutic approach for enzymatic LSDs, emerging evidence indicates that promoting lysosomal membrane trafficking could be another treatment for both enzymatic LSDs and LSDs associated with dysfunctional lysosome membrane proteins^3^,^4^,^5^,^6^,^7^.

Transient Receptor Potential Mucolipin-1 (TRPML1) is a well-known lysosomal Ca^2+^ release channel that is essential for the regulation of lysosomal membrane trafficking. TRPML1 deficiency results in enlarged lysosomes and abnormal lysosomal storage, as shown in cells from the patients with mucolipidosis type IV (ML4), a LSD caused by loss-of-function mutations of human TRPML1^8^,^9^. Recently, we have shown that TRPML1 forms a macromolecular complex with BK in the lysosome membrane where BK is activated by Ca^2+^ released via TRPML1. Activated BK provides K^+^ influx to facilitate TRPML1 opening by maintaining a lysosomal membrane potential beneficial for TRPML1. Therefore, BK and TRPML1 forms a positive feedback loop to facilitate lysosomal Ca^2+^ release and subsequent lysosomal membrane trafficking and function^7^.

Defective lysosomal membrane trafficking is commonly seen in LSDs^4^,^5^,^10^,^11^,^12^,^13^. Although abnormal TRPML1 has been associated with a number of LSDs including ML4, Niemann Pick type C1 (NPC1), NPA and NPB^10^,^11^,^12^,^13^,^14^, it remains elusive whether BK is also implicated in these diseases and whether BK agonists could be potential therapeutic drugs. In this study, we aim to test whether boosting BK channel function corrects the lysosome defects in LSDs. In particular, we assessed the effect of BK channel agonists, on TRPML1 channel activity and the clearance of lysosomal storage in human fibroblasts from NPC1, mild cases of ML4, NPA and Fabry disease^3^,^4^,^8^. Our data have suggested that activating BK channels facilitates TRPML1-mediated lysosomal Ca^2+^ release, and subsequently alleviates the cellular phenotypes of these LSDs by promoting lysosomal exocytosis. Thus, promoting BK activity could be a common therapeutic approach to cure LSDs.

## Results

### NS1619 rescues NPC1 phenotypes via promoting TRPML1-dependent lysosomal exocytosis

**Figure 1:**
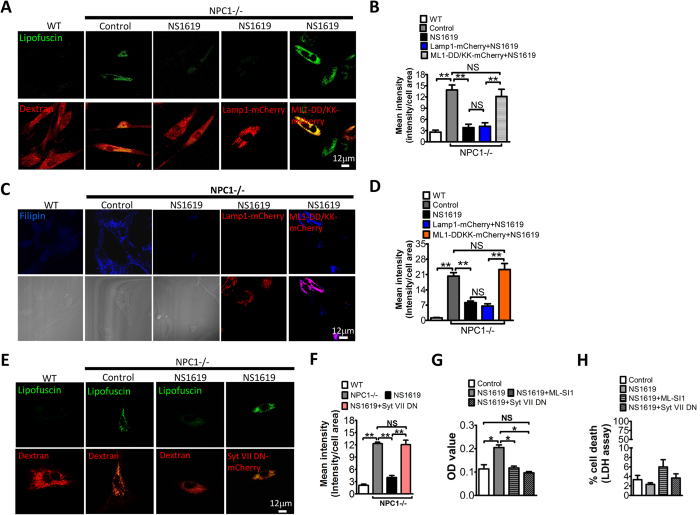
Activation of BK by NS1619 reduces lipofuscin and cholesterol accumulation in NPC1 cells in a TRPML1-dependent manner. (**A,B**) Abnormal lipofuscin accumulation (detected by autofluorescence) in NPC1 human fibroblasts and its rescue by activation of BK with NS1619 (15 μM, 16 hrs). Expression of TRPML1-DD/KK reversed the rescue effect of BK activation. In some groups, dextran staining was employed to indicate the presence of cells. More than 42 cells were analyzed for each condition. (**C,D**) Abnormal cholesterol accumulation (filipin staining) in NPC1 human fibroblasts and its rescue by activation of BK with NS1619 (15 μM, 18 hrs). Expression of TRPML1-DD/KK reversed the rescue effect of BK activation. In some groups, bright field images were included to indicate the presence of cells. More than 41 cells were analyzed for each condition. (**E,F**) NS1619 treatment (15 μM, 16 hrs) reduced the abnormal lipofuscin accumulation in NPC1 human fibroblasts. This NS1619 effect was inhibited by overexpressing Syt VII DN that suppresses lysosomal exocytosis. (**G**) NS1619 (15 μM, 16 hrs) increased lysosomal exocytosis (indicated by the elevation of lysosomal enzyme β-hexosaminidase in cell culture supernatant), which was inhibited by applying ML-SI1 or expressing Syt VII DN. (**H**) Comparable LDH in culture medium under conditions indicated, suggesting the elevation of β-hexosaminidase in culture media was not attributed to cell death which releases lysosomal enzymes. The data represents mean ± SEM, and experiments were repeated independently 3 times in triplicate.

NPC1 disease is caused by defects in the gene NPC1 that transports cholesterol across lysosomal membrane. NPC1 mutations result in impaired lysosomal membrane trafficking, leading to abnormal lipofuscin, cholesterol and sphingomyelin accumulation in lysosomes^4^,^15^. Recent studies have suggested that TRPML1-mediated Ca^2+^ release is compromised in NPC1 human fibroblasts^4^,^7^. We have shown that BK overexpression facilitates TRPML1 function, thereby rescuing abnormal lysosomal storage in NPC1 human fibroblasts^7^. To help develop a potential therapeutic strategy to cure NPC1 by potentiating BK, we aim to study whether NS1619, a chemical reported to activate BK in the plasma membrane (PM)^16^, eliminates NPC1 cellular phenotypes. As shown in Fig. 1A, human skin fibroblasts from NPC1 patients showed significantly higher signal of lipofuscin, a non-degradable auto-fluorescent polymeric substance often accumulated with age or seen in LSDs and aging diseases^7^,^8^,^17^,^18^. This was inhibited by NS1619 treatment in a time and does-dependent manner (Fig. S1A,B). Additionally, the abnormal lipofuscin in NPC1 human fibroblasts could be rescued by NS11021 (Fig. S1C), another BK agonist with high potency and selectivity^19^. Interestingly, the inhibitory effect of NS1619 (15 μM, 16 h) on lipofuscin signal in NPC1 fibroblasts was eliminated by TRPML1-DD/KK, a dominant negative mutant of TRPML1^20^,^21^, but not Lamp1 (Fig. 1A,B). These data suggest that NS1619 corrects lipofuscin accumulation in NPC1 cells through promoting TRPML1 activity. Similarly, NS1619 (15 μM, 18 h) rescued cholesterol accumulation in NPC1 cells using a TRPML1-dependent mechanism (Fig. 1C,D).

Promoting lysosomal exocytosis (lysosome fusion with the plasma membrane) is known as a promising therapeutic approach to attenuate lysosomal storage and treat LSDs^3^,^4^,^5^,^6^. Emerging evidence indicates that TRPML1 is important for lysosomal exocytosis^6^,^22^,^23^,^24^. The rescue effect of NS1619 on lipofuscin could be attributed to an enhanced lysosomal exocytosis. To test this possibility, NPC1 fibroblasts were expressed with Syt VII DN, a dominant-negative (DN) form of synaptotagmin VII^24^,^25^ that inhibits lysosomal exocytosis. As shown in Fig. 1E,F, NS1619’s rescue effect on lipofuscin in NPC1 cells was inhibited by expressing Syt VII DN. To directly evaluate the role of NS1619 in lysosomal exocytosis, the level of lysosomal enzyme β-hexosaminidase in culture media was monitored. NS1619 treatment significantly increased the release of β-hexosaminidase in NPC1 fibroblasts, and this was suppressed by co-applying ML-SI1, an TRPML1 channel inhibitor^7^, or expressing Syt VII DN (Fig. 1G). The increase of lysosomal β-hexosaminidase in culture media could be due to cell death. To exclude this possibility, we thus measured the levels of lactate dehydrogenase (LDH) to monitor the cell death^26^. Neither ML-SI1 nor Syt VII DN significantly changed the LDH level in culture media (Fig. 1H). Taken together, we suggest that NS1619 rescues NPC1 cellular phenotypes by promoting TRPML1-mediated lysosomal exocytosis.

### NS1619 directly activates BK channels but not TRPML1 in lysosomes

**Figure 2:**
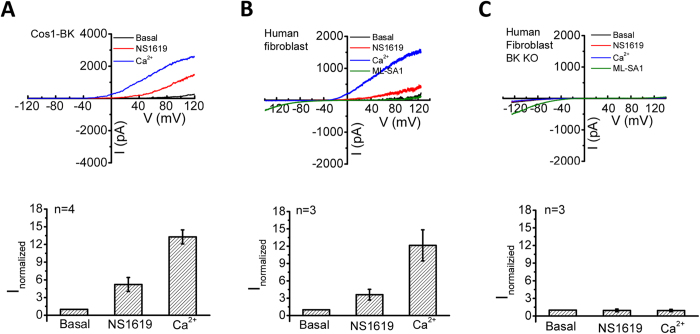
Activation of BK channels by NS1619. (**A**) Bath application of NS1619 (15 μM), a BK activator, significantly increased BK currents, but not inwardly rectifying ML1 currents in lysosomes isolated from Cos1 cells expressing BK-GFP. Bath application of 100 μM Ca^2+^ was employed to maximally activate BK currents. (**B**) NS1619 (50 μM) markedly increased outwardly rectifying BK currents but not inwardly rectifying ML1 currents in lysosomes isolated from human skin fibroblasts. ML-SA (20 μM) was used to induce TRPML1 currents. (**C**) Bath NS1619 (50 μM) did not induce outward current in lysosomes isolated from BK knockout human fibroblasts, whereas ML-SA (20 μM) induced significant TRPML1 currents. (Upper) representative BK currents in response to a voltage ramp (from −120 mV to 120 mV, v_holding_ = 0 mV). (Bottom) Normalized currents (normalized to the basal current amplitude under 100 nM Ca^2+^) measured at 105 mV.

The rescue effect of NS1619 on NPC1 cellular phenotypes could be attributed to activating either BK or TRPML1. To tease apart these two possibilities, we directly studied NS1619’s effect on lysosomal BK and TRPML1 channel activity. Under whole-lysosome recording mode, TRPML1 displays strongly inward rectification while BK shows strongly outward rectification^7^. In Cos1 cells expressing BK-GFP, 15 μM NS1619 significantly increased lysosomal BK currents by ~5 fold (at 105 mV) (Fig. 2A). In wild type human skin fibroblasts, 50 μM NS1619 also significantly increased endogenous lysosomal BK currents by ~4 fold (at 105 mV) (Fig. 2B). In contrast, NS1619 (50 μM) induced little BK current in human fibroblasts with BK deleted using CRISPR-Cas9 gene editing method^7^ (Fig. 2C), suggesting that NS1619 specifically activates BK to induce the outwardly rectifying currents. On the other hand, NS1619 (50 μM) had no effect on both endogenous (Fig. 2B,C) and heterologous (data not shown) TRPML1 currents, which could be activated by the ML1 agonist, mucolipin synthetic agonist 1 (ML-SA1).

### NS1619 facilitates TRPML1-mediated Ca^2+^ release

**Figure 3:**
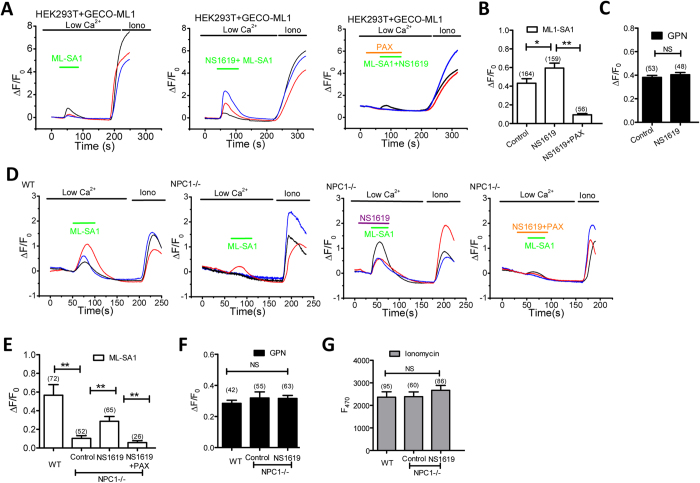
Activation of BK by NS1619 facilitates ML1-mediated Ca^2+^ release. (**A,B**) NS1619 (15 μM) treatment increased ML-SA1 (10 μM)-mediated GECO-TRPML1 response, which was inhibited by co-applying Paxilline (PAX, 3 μM) in HEK293T cells. (**C**) NS1619 (15 μM) treatment did not alter GECO-TRPML1 response to GPN (200 μM), suggesting lysosomal Ca^2+^ content was not affected. (**D,E**) NPC1 human fibroblasts exhibited impaired GECO-TRPML1 response to ML-SA1 (10 μM). NS1619 (15 μM) treatment increased GECO-TRPML1 response to ML-SA1, and this was inhibited by co-applying Paxilline (PAX, 3 μM). (**F**) GECO-TRPML1 responses to GPN (200 μM) was not altered by NS1619 (15 μM) in NPC1 cells, suggesting that NS1619 does not change lysosomal Ca^2+^ content. (**G**) GECO-TRPML1 responses to Ionomycin (1 μM) were not altered, indicating a similar level of GECO-TRPML1 expression.

We have shown that BK activation facilitates TRPML1 function by maintaining a beneficial lysosomal membrane potential for TRPML1^7^. To test whether the beneficial effect of NS1619 in NPC1 is dependent on TRPML1 activation, we tested whether NS1619 increases TRPML1-mediated Ca^2+^ release using GECO-TRPML1, a single-wavelength genetically encoded Ca^2+^ indicator^7^,^27^. In cells bathed in a low Ca^2+^ (free Ca^2+^ < 10 nM) solution, an increase in GECO-TRPML1 signal which is monitored by measuring GECO fluorescence (F470) represents TRPML1 activation. GECO-TRPML1 was expressed in HEK293T cells, and GECO-TRPML1 fluorescence in response to ML-SA1 was compared between NS1619 and control vehicle. As shown in Fig. 3A,B, NS1619 (15 μM) significantly increased GECO-TRPML1 fluorescence signal in response to ML-SA1, as compared to the control. This was suppressed by Paxilline (PAX), the selective BK channel blocker^7^. Notably, NS1619 did not affect lysosomal Ca^2+^ content, because glycyl-phenylalanine 2-naphthylamide (GPN, 200 μM), a substrate of the lysosomal exopeptidase cathepsin C that induces lysosome osmolysis to deplete lysosomal Ca^2+^ pools^4^, induced a comparable GECO-ML1 response between the control and NS1619 treatment (Fig. 3C). These data suggest that NS1619 facilitates lysosomal Ca^2+^ release through TRPML1.

Second, we tested whether NS1619 increases TRPML1-mediated Ca^2+^ release in NPC1 cells. As shown previously^4^,^15^, the ML-SA1-evoked GECO-TRPML1 response was decreased in NPC1 fibroblasts (Fig. 3D,E). Interestingly, NS1619 partially rescued the impaired GECO-TRPML1 response, whereas the lysosomal Ca^2+^ content was not affected (Fig. 3D–F). These data suggest that activating BK promotes TRPML1-mediated Ca^2+^ release in NPC1 fibroblasts.

### Up-regulation of BK channels promotes lysosomal Ca^2+^ release in TRPML1 deficient cells

**Figure 4:**
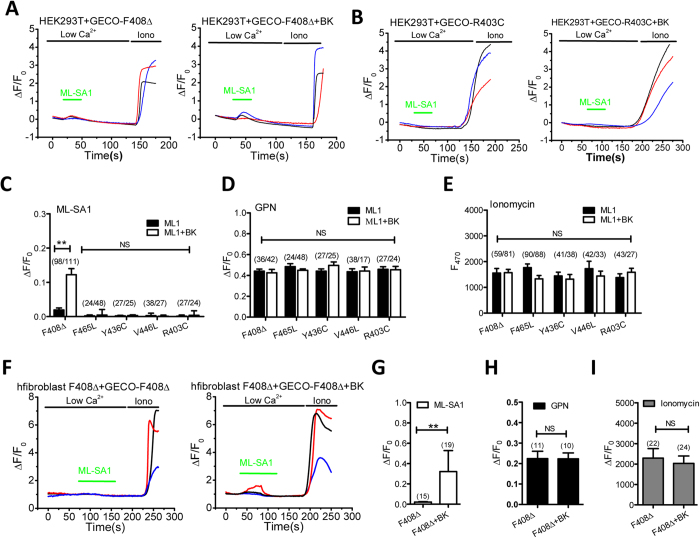
BK upregulation promotes lysosomal Ca^2+^ release in ML4 fibroblasts carrying F408Δ mutation. (**A**) BK overexpression increased ML-SA1 (10 μM)-induced GECO-TRPML1-F408Δ response in HEK293T cells. (**B**) BK overexpression did not alter ML-SA1 (10 μM)-induced GECO-TRPML1-R403C response in HEK293T cells. (**C**) Statistical analysis of GECO response to ML-SA1 (10 μM) showing that BK overexpression significantly increased GECO-TRPML1-F408Δ signal but not others. (**D**) BK overexpression did not alter GPN (200 μM)-induced GECO responses. (**E**) BK overexpression did not alter Ionomycin (1 μM)-induced GECO responses. (**F,G**) BK overexpression enhanced GECO-TRPML1-F408Δ response to ML-SA1 (10 μM) in TRPML1-F408Δ human fibroblasts, suggesting that BK upregulation facilitates TRPML1-F408Δ activity. (**H,I**) GECO-TRPML1-F408Δ responses to GPN (200 μM) (**H**) and Ionomycin (1 μM) (**I**) were not altered in TRPML1-F408Δ human fibroblasts by BK overexpession, suggesting BK overexpression did not alter lysosomal Ca^2+^ content and GECO- TRPML1-F408Δ expression level in TRPML1-F408Δ human fibroblasts, respectively.

Although all ML4 patients are characterized by motor impairment, mental retardation, and retinal degeneration, the severity of the phenotypes are different depending on the type of mutation^3^,^8^. Some mutations result in complete absence of TRPML1 or nonfunctional TRPML1, such as V446L, D362Y and T232P. Some mutations affect the ion conducting pore, leading to compromised channel activity, such as F408∆, F465L and R403C^8^. Some mutations appear to affect localization of the proteins, such as L447P, V446L, R403C and D362Y^3^. Mutant TRPML1 isoforms that remain correctly localized but may be largely compromised could be good candidates for a small molecule therapeutic approach, such as F408∆^3^,^8^. Given that BK overexpression^7^ or activation (Fig. 3) facilitates TRPML1 activity, we speculate that upregulating BK may rescue phenotypes of cells with compromised TRPML1 activity but relatively correct localization (F408∆). To test this hypothesis, we introduced five ML4 disease-related mutations (F408∆, F465L, R403C, Y436C, V446L) into the GECO-TRPML1, and then tested whether BK overexpression or activation increases mutant GECO-TRPML1 response in intact cells. We observed a significant GECO signal in F408∆ but not other ML4 mutations (R403C, F465L, Y436C and V446L) in response to ML-SA1 (Fig. 4A–C). The difference in GECO signal observed in these ML4 mutants was not caused by a change in lysosomal Ca^2+^ content (Fig. 4D) or in GECO overexpression level, which was indicated by the maximal fluorescence increase induced by ionomycin (1 μM, with 2 mM Ca^2+^ in the bath)^4^ (Fig. 4E). In agreement with these, patients carrying F408∆ mutation have mild phenotypes whereas those carrying R403C, F465L, Y436C, V446L mutations have severe phenotypes^8^. Interestingly, the compromised GECO response to ML-SA1 in GECO-TRPML1-F408∆ was partially corrected by BK overexpression (Fig. 4A,C). Consistently, BK overexpression significantly increased GECO response to ML-SA1 in F408∆ patient fibroblasts transfected with GECO-TRPML1-F408∆ (Fig. 4F,G), while lysosomal content (Fig. 4H) and GECO-TRPML1-F408∆ overexpression (Fig. 4I) were not altered by BK overexpression. These results suggest that BK potentiates the activity of TRPML1 carrying F408∆ mutation that exhibits relatively significant channel activity and correct localization. This effect was not seen in other ML4 mutants that are either dysfunctional or mis-targeted.

### Upregulating BK channels corrects abnormal lysosomal storage in ML4 (F408∆) human fibroblasts

**Figure 5:**
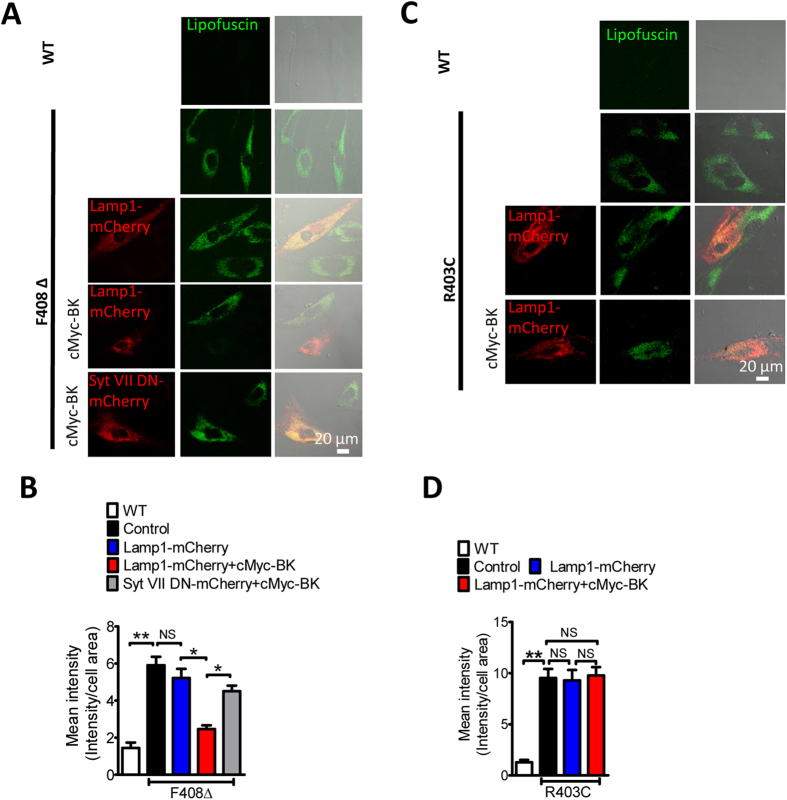
BK upregulation reduces abnormal lipofuscin accumulation in human fibroblasts carrying F408∆ but not R403C mutation. (**A,B**) Abnormal lipofuscin accumulation in TRPML1-F408∆ human fibroblasts and its rescue by BK overexpression. The BK rescue effect was inhibited by Syt VII DN, suggesting it depends on promoting lysosomal exocytosis. More than 35 cells were analyzed for each condition. (**C,D**) Abnormal lipofuscin accumulation in TRPML1-R403C human fibroblasts was not rescued by BK overexpression. More than 40 cells were analyzed for each condition.

Defective TRPML1 channels results in abnormal lysosomal storage, which often leads to the formation of autofluorescent lipofuscin, as seen in LSD human skin fibroblasts^4^,^8^. Not surprisingly, a dramatic increase in lipofuscin fluorescence was observed in human fibroblasts carrying F408∆ or R403C mutation (Fig. 5). BK overexpression significantly reduced the intensity of autofluorescence in F408∆ (Fig 5A,B), but not in R403C fibroblasts (Fig. 5C,D). Expression of Syt VII DN prohibited the rescue effect of BK overexpression on lipofuscin accumulation in F408∆ fibroblasts (Fig. 5A,B). Considering that BK upregulation promotes GECO-TRPML1-F408∆ signal, these data indicate that BK up-regulation enhances TRPML1-F408∆-mediated lysosomal exocytosis, and thereby promoting lipofuscin clearance from F408∆ fibroblasts.

### Upregulation of BK channels rescues NPA phenotypes

**Figure 6:**
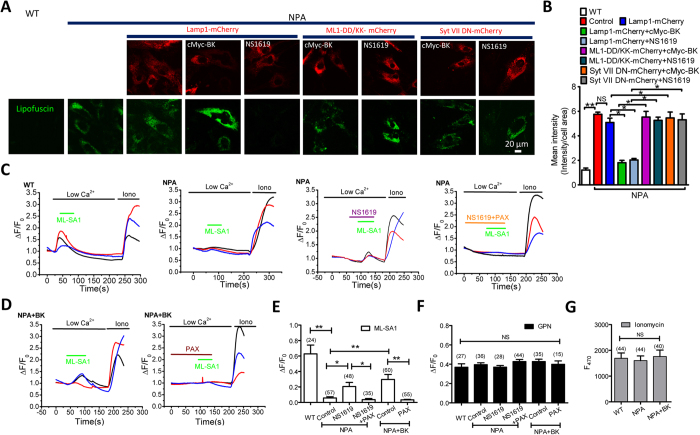
BK upregulation reduces lipofuscin accumulation in NPA cells by promoting TRPML1-mediated Ca^2+^ release. (**A,B**) Abnormal lipofuscin accumulation in NPA human fibroblasts and its rescue by BK overexpression or NS1619 (15 μM, 16 h) treatment. Expression of TRPML1-DD/KK or Syt VII DN reversed the rescue effect of BK upregulation. More than 35 cells were analyzed for each condition. (**C–E**) Impaired ML-SA1 (10 μM)-mediated GECO-TRPML1 response in NPA human fibroblasts and its rescue by NS1619 (15 μM, ~60 s) pretreatment (**C**) or BK overexpression (**D**). Paxilline (PAX, 3 μM) treatment reversed the rescue effect of NS1619 or BK overexpression. (**F,G**) GECO-TRPML1 responses to GPN (200 μM) (F) and Ionomycin (1 μM) (**G**) were not altered in all the treatments, suggesting lysosomal Ca^2+^ content, or GECO-TRPML1 expression level was not affected.

In Niemann-Pick type A (NPA) and type B (NPB) diseases, sphingomyelin accumulation in lysosomes due to insufficient activity of acid sphingomyelinase results in reduced TRPML1-mediated lysosomal Ca^2+^ release and lysosomal storage^4^,^4^,^10^,^11^,^15^. Given that BK promotes TRPML1-mediated lysosomal Ca^2+^ release, we hypothesize that BK upregulation rescues NPA phenotypes. As expected, NPA cells displayed increased lipofuscin autofluoresence, as compared to wild type human fibroblasts^4^,^15^, and this was rescued by BK overexpression or NS1619 (15 μM, 16 h) treatment (Fig. 6A,B). Expression of TRPML1-DD/KK or Syt VII DN inhibited the rescue effect of BK. Therefore, BK upregulation rescues lysosomal storage in NPA patient cells through facilitating TRPML1-mediated lysosomal exocytosis.

To confirm that the rescue effect of BK in NPA cells is dependent on TRPML1 activation, we measured TRPML1-mediated Ca^2+^ release in NPA cells. As shown in Fig. 6C–E, the ML-SA1-mediated GECO-TRPML1 response was decreased in NPA fibroblasts. Either NS1619 or BK overexpression partially rescued the impaired GECO-TRPML1 response, whereas the lysosomal Ca^2+^ content (Fig. 6F) and GECO-TRPML1 expression level (Fig. 6G) was not affected. Altogether, these data suggest that BK upregulation promotes TRPML1-mediated Ca^2+^ release in NPA patient fibroblasts, thereby correcting the impaired lysosome trafficking and storage in these cells.

### Upregulation of BK channels rescues the cellular phenotypes of Fabry disease

**Figure 7:**
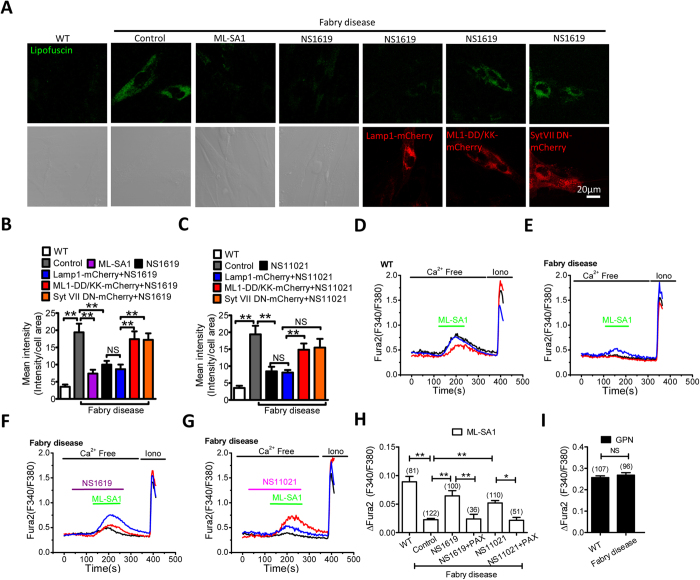
BK upregulation reduces lipofuscin accumulation in human skin fibroblasts from Fabry disease by promoting TRPML1-mediated Ca^2+^ release. (**A,B**) Abnormal lipofuscin accumulation in human fibroblasts from Fabry disease was rescued by ML-SA1 (10 μM, 16 hrs) or NS1619 (15 μM, 16 hrs). Expression of TRPML1-DD/KK or Syt VII DN reversed the rescue effect of ML-SA1 and NS1619. More than 40 cells were analyzed for each condition. (**C**) Abnormal lipofuscin accumulation in human fibroblasts from Fabry disease was rescued by NS11021 (3 μM, 16 hrs). Expression of TRPML1-DD/KK or Syt VII DN reversed the rescue effect of NS11021. More than 40 cells were analyzed for each condition. (**D–H**) human fibroblasts from Fabry disease exhibited compromised TRPML1-mediated lysosomal Ca^2+^ release in response to ML-SA1 (50 μM) (as measured by Fura2 ratio), and this was rescued by NS1619 (15 μM, ~60 s) or NS11021 (3 μM, ~ 60 s) pretreatment. Co-applying Paxilline (PAX, 3 μM) inhibited the rescue effect of NS1619 or NS11021. (**I**) Comparable Fura2 responses to GPN (200 μM) between human fibroblasts from Fabry disease and WT human fibroblasts, indicating a similar level of lysosomal Ca^2+^ content.

All LSD models used above have been shown to display a compromised TRPML1 activity. To test whether BK therapy could be a general treatment for the majority of LSDs, we aimed to assess the effect of BK agonists on another LSD model where TRPML1 function has not been measured. Fabry disease is an inherited LSD involving dysfunctional metabolism of sphingolipids. The disease accumulates storage material and leads to a wide range of systemic symptoms including kidney, heart, dermatological and neurologic defects^28^,^29^. As shown in Fig. 7A, human skin fibroblasts from Fabry disease patients (GM00107)^28^ displayed a much higher level of lipofuscin autofluoresence compared to wild type human fibroblasts, and this was decreased by activating TRPML1 with ML-SA1 (15 μM, 16 h), and by activating BK with NS1619 (15 μM, 16 h) or NS11021 (3 μM, 16 h) (Fig. 7 A–C), respectively. The rescue effect of NS1619 or NS11021 was abolished by expressing TRPML1-DD/KK or Syt VII DN, suggesting that BK upregulation-mediated lipofuscin reduction is dependent on TRPML1-mediated lysosomal exocytosis. In agreement with this, we found that TRPML1 activity was inhibited in cells from Fabry disease patient, as indicated by a decrease in TRPML1-meidated lysosomal Ca^2+^ release in respond to ML-SA1 (Fig. 7D,E,H). In addition, NS1619 (15 μM) or NS11021 (3 μM) rescued TRPML-mediated lysosomal Ca^2+^ release, and this was abolished by inhibiting BK with Paxilline (3 μM) (Fig. 7F,G,H). Notably, GPN-induced lysosomal Ca^2+^ release was comparable, indicating a similar level of lysosomal Ca^2+^ content between cells from Fabry disease patient and WT cells (Fig. 7I). Taken together, these data suggest that BK upregulation reduce lipofuscin accumulation in Fabry disease via increasing TRPML1-mediated lysosomal exocytosis.

## Discussion

The lysosome acts as a garbage dump or recycling center inside of the cell. Dysfunction of the lysosome causes accumulation of waste products in the cell, eventually leading to cell death which is manifested by a large number of inherited metabolic disorders, called lysosomal storage diseases (LSDs). In general, LSDs are subdivided into two groups. One subgroup of LSDs is caused by deficiency of specific enzymes that are normally required for the breakdown of certain complex macromolecules including carbohydrates, lipids, proteins, and nucleic acids. The other subgroup of LSDs result from some defective lysosome membrane proteins which are essential for creating optimal environment for lysosomal enzymes, transporting metabolites, or lysosomal membrane trafficking^1^,^2^,^5^,^11^,^12^,^13^,^30^. Regardless of the nature of the primary causes, defective membrane trafficking is common in LSDs^1^,^2^,^5^,^11^,^12^,^13^,^15^,^30^. Recent studies suggest that promoting lysosomal membrane trafficking, particularly lysosomal exocytosis, represents a promising therapeutic approach for both enzymatic LSDs and LSDs associated with dysfunctional lysosome membrane proteins^3^,^4^,^5^,^6^. As a ubiquitously expressed lysosomal Ca^2+^ release channel that regulates lysosomal membrane trafficking^7^,^31^, TRPML1 has been implicated in a number of LSDs^4^,^7^.

We have previously demonstrated that BK forms a macromolecular complex with TRPML1 in lysosomes to ensure sufficient lysosomal Ca^2+^ release and membrane trafficking using a positive feedback mechanism. In this study we further strengthen this notion using a number of LSD models. We found that upregulating BK rescued the phenotypes of patient fibroblasts from NPC1, F408∆-ML4, NPA and Fabry disease by promoting lysosomal Ca^2+^ release and subsequent lysosomal exocytosis. Therefore, in certain types of LSDs, activating BK channels strengthens the positive feedback loop between TRPML1 and BK, and subsequently alleviates the diseases^3^,^4^,^8^. Indeed, in a translational approach, we studied the potential of BK channel agonists in rescuing TRPML1 channel activity as well as abnormal lysosomal storage in these LSDs. We found that BK upregulation has the ability to restore TRPML1-mediated lysosomal Ca^2+^ release and to clear lysosomal storage in skin fibroblasts from a number of LSDs by improving lysosomal exocytosis. Given that TRPML1 is a nonselective cation channel that permeates both Ca^2+^ and Na^+ ^8, the Na^+^ efflux could also carry some important functions, such as membrane potential regulation and Na^+^-dependent solute transport, to correct the cellular phenotypes of these LSDs. The demonstration that BK helps clear lysosome storage in these LSD models would argue that Ca^2+^ but not Na^+^ efflux represents the main cellular function of the TRPML1 channel in rescuing LSDs. This is further supported by the finding that the rescue effect of upregulating either TRPML1^4^ or BK^7^ is eliminated by disrupting the lysosomal Ca^2+^ sensor using Syt VII DN. Collectively, our data demonstrate that small molecules targeting on BK channels can be used to restore lysosomal function and rescue disease associated abnormalities in patient cells with lysosomal storage. This may lead to a new clinical intervention strategy for treating LSDs in the future.

In addition to LSDs^3^,^4^,^5^,^6^,^8^,^11^,^12^,^15^, classical forms of neurodegenerative diseases have been associated with abnormal lysosomal Ca^2+^ release and membrane trafficking^14^,^32^,^33^,^34^,^35^,^36^,^37^,^38^. In particular, TRPML1 has been implicated in Aβ clearance from lysosomes^38^. Therefore, this study will also guide the design of new therapeutics by identifying novel chemicals as drugs that target BK to cure a number of neurodegenerative diseases including Parkinson’s disease, Alzheimer’s disease, Huntington’s disease, and Amyotrophic Lateral Sclerosis.

## Experimental Procedures

### Cell culture

Cos1 cells and HEK293T cells were obtained from ATCC (Manassas, VA) and maintained in Dulbecco’s Modified Eagle’s Medium: Nutrient Mixture F-12 (DMEM/F12) supplemented with 10% fetal bovine serum (FBS, Invitrogen, Carlsbad, CA, USA). Human skin fibroblasts from NPC1 patient (NPC1, GM03123), NPA (GM00112) ML4 patient (GM02629), Fabry disease patient (GM00107) and non-disease control cells (GM00969) were obtained from Coriell Institute for Medical Research (NJ, USA). Human fibroblasts carrying ML4 mutations (F408∆, R403C) were from Baylor Institute of Metabolic Disease. All the fibroblasts were maintained in DMEM supplemented with 15% non-heat-inactivated FBS. Cells were cultured at 37˚C in a 5% CO_2_ atmosphere. For some experiments, cells were seeded on 0.01% Poly-D-Lysine coated glass coverslips and cultured for 24 hrs before further experimentation. Cos1 and HEK293T cells were transiently transfected with indicated DNA using Lipofectamine 3000^®^ following manufacturer’s manual. Fibroblasts were transiently transfected with indicated DNA by Neon^®^ electroporation (100 μl tip, 1450 V ~ 1550 V, 28 ms, 1 pulse) and cultured for 24 hrs before use.

### Reagents

The following chemicals were used: Texas Red 10 kD dextran (Invitrogen, 1 mg/ml); NS1619 (Sigma); NS11021 (Sigma); GPN (Santa Cruz Biotechnology); Paxilline (Cayman Chemical Company); ML-SA1 (Tocris); Ionomycin (Cayman), ML-SI1 (Enzo Life Sciences Inc).

### Molecular biology

Plasmids cMyc-BK-GFP constructs were prepared as before^39^. The GECO-TRPML1 construct was made by inserting the full length GECO sequence^27^ between the HindIII and BamHI sites of a pcDNA6 plasmid that contains the mouse TRPML1 cDNA at the XhoI site. GECO-TRPML1-F408Δ, GECO-TRPML1-F465L, GECO-TRPML1-Y436C, GECO-TRPML1-V446L and GECO-TRPML1-R403C and TRPML1 non-conducting pore mutant (D471K/D472K; abbreviated TRPML1-DD/KK) were constructed using a site-directed mutagenesis kit (Qiagen). Syt VII DN-mCherry was made by amplifying Syt VII DN cDNA from dominant negative pShooter-Flag-Syt VII-D172N/D303N-GFP that was generously provided by Mitsunori Fukuda, and then the Syt VII DN cDNA was inserted between NheI and HindIII site of mCherry-N1 vector. TRPML1-mCherry and TRPML1-DD/KK-mCherry were made as described before^8^,^24^. Lamp1-mCherry was a gift from Michael X. Zhu.

### Knockout of human BK using CRISPR/Cas9 system

The operation followed detailed instructions of the published protocol^40^. In brief, 20-bp target single-guide RNA sequences (gRNA) were obtained by screening BK mRNA sequence with CRISPR DESIGN online software (http://crispr.mit.edu/), and two sequences were chosen: CATCTTGGGCTCGTGGACCG (target 1) and CTAGGCTGAGATGGTTCGCG (target 2). After being annealed with their reverse complementary sequences, the short double strand DNA’s (hereafter named gDNA) were ligated with *Bbs I* digested pX330-2A-GFP and the new constructs were named BK-CRISPR1 and 2, respectively. Human skin fibroblasts were transfected with individual pX330-2A-GFP/BK gDNAs using Neon^®^ system following optimized protocol and cultured for 72 hours before use. The transfection efficiency for human fibroblasts was >70%.

### Lysosomal electrophysiology

Lysosomal electrophysiology was performed in isolated enlarged late endosome/lysosome vacuoles using whole-lysosome patch clamp method as described previously^8^. Briefly, cells were treated with 1 μM vacuolin-1, a lipophilic polycyclic triazine that can selectively increase the size of endosomes and lysosomes, for 2–6 hrs. Enlarged vacuoles were manually isolated and recording under whole-lysosome mode. Unless otherwise stated, bath (cytoplasmic) solution contained 140 mM K-gluconate, 4 mM NaCl, 1 mM EGTA, 2 mM MgCl_2_, 0.39 mM CaCl_2_, 20 mM HEPES (the pH was adjusted with KOH to 7.2; free [Ca^2+^] was 100 nM calculated using the Maxchelator software). The pipette (luminal) solution was a standard extracellular solution contained 145 mM NaCl, 5 mM KCl, 2 mM CaCl_2_, 1 mM MgCl_2_, 10 mM HEPES, 10 mM glucose (the pH was adjusted with HCl to 4.6). Whole-lysosome currents were digitized at 10 kHz and filtered at 2 kHz. Liquid junction potential was corrected. Data were collected using an Axopatch 2A patch-clamp amplifier, Digidata 1440, and pClamp 10.2 software (Axon Instruments). All experiments were conducted at room temperature (21 °C–23 °C).

### GECO Ca^2+^ imaging

HEK293T cells or human fibroblasts were transfected with GECO-TRPML1 for 24–36 hrs and then plated onto glass coverslips. Imaging was carried out within 0.5–2 hrs after plating when cells still exhibited a round morphology. The fluorescence intensity at 470 nm (F470) was recorded using the EasyRatioPro system (PTI). Lysosomal Ca^2+^ release was measured under a ‘low’ external Ca^2+^ solution that contained 145 mM NaCl, 5 mM KCl, 3 mM MgCl_2_, 10 mM glucose, 1 mM EGTA and 20 mM HEPES (pH was adjusted to 7.4 using NaOH, free [Ca^2+^] <10 nM calculated using Maxchelator software. Experiments were conducted at room temperature (21 °C–23 °C), and repeated three times independently.

### Fura-2 Ca^2+^ imaging

Cells were loaded with 5 μM Fura-2 AM (Invitrogen). Fluorescence was recorded at different excitation wavelengths using an EasyRatioPro system (PTI). Fura-2 ratios (F_340_/F_380_) were used to monitor changes in intracellular [Ca^2+^] upon stimulation. Lysosomal Ca^2+^ release was measured under a ‘low’ external Ca^2+^ solution. ML-SA1 (50 μM) and GPN (400 μM) were used to induce Ca^2+^ release from lysosomes. Ionomycin (1 μM) was added at the conclusion of all experiments to induce a maximal response for comparison. Ca^2+^ imaging was carried out within 0.5–2 h after plating and when cells still exhibited a round morphology.

### Filipin Staining

Cells were fixed with 3% paraformaldehyde for 1 hr and washed three times with PBS. Fixed cells were then incubated with 1.5 mg/ml glycine in PBS for 15 min at room temperature to quench the paraformaldehyde. After three washes with PBS, cells were stained with filipin (Sigma, 0.05 mg/ml with 10% FBS in PBS) for 2 hrs at room temperature. Cells were rinsed three times with PBS and viewed with Zeiss LSM710 confocal microscope (40 × or 63× oil-immersion objective lens) using 340–380 nm excitation and 510 nm emission.

### Confocal microscopy

Confocal fluorescent images were taken using an inverted Zeiss LSM510 Axiovert 200M confocal microscope with a 63× oil-immersion objective. Sequential excitation wavelengths at 488 nm and 543 nm were provided by argon and helium-neon gas lasers, respectively. Emission filters BP500-550 and LP560 were used for collecting green and red images in channels one and two, respectively. After sequential excitation, green and red fluorescent images of the same cell were saved with ZEN2012 software. In some experiments, autofluorescence (green) detectable within the excitation wavelength of 480 nm is an indicator of lipofuscin accumulation. Images were analyzed by Zeiss software. Intensity of fluorescent was analyzed using ImageJ, The image size was set at 1,024 × 1,024 pixels.

### β-Hexosaminidase assay and LDH assay

NPC1 cells were cultured in 24-well plates. β-hexosaminidase activity in 100 μl supernatant samples was measured by incubation with 100 μl of 1 mM *p*-nitrophenyl *N*-acetyl-*β*-*D*-glucosaminide in 0.1 M citrate buffer (0.05 M citric acid, 0.05 M sodium citrate, pH 4.5) for 1 hour at 37 °C. Reactions were quenched by addition of 200 μl 0.1 M sodium carbonate buffer. Absorbance was read at 405 nm. Absorbance values were background subtracted using medium only values. LDH activity was determined by measuring the NADH oxidation with pyruvate as substrate. For each assay, a 50 μl sample was incubated with 50 μl reaction buffer (CytoTox-ONE™ Homogeneous Membrane Integrity Assay, Promega) for 10 min at 22 °C and the fluorescence at 340 nm was measured using a spectrophotometer. Results were scaled to the complete cell death induced by exposure to 1% TritonX100 at room temperature for 20 min. Percent of cell death was defined as = 100 × (sample’s OD - background medium OD)/(Triton-X100 OD - medium background OD).

### Data analysis

Experiments were repeated at least three times independently. Data are presented as mean ± SEM. Analysis of variance (ANOVA) or Student’s t test was applied for statistical comparisons. P values of <0.05 were considered statistically significant. NS: not significant; *P < 0.05; **P < 0.01.

## Additional Information

**How to cite this article**: Zhong, X. Z. *et al*. BK channel agonist represents a potential therapeutic approach for lysosomal storage diseases. *Sci. Rep.* **6**, 33684; doi: 10.1038/srep33684 (2016).

## Supplementary Material

Supplementary Information
