# Supplementary material for: BK channel agonist represents a potential therapeutic approach for lysosomal storage diseases
Source: Sci Rep. 2016 Sep 27;6:33684. doi: 10.1038/srep33684 (PMC5037385; doi:10.1038/srep33684)
Supplement: Supplementary Information [file srep33684-s1.pdf]

BK channel agonist represents a potential therapeutic approach for lysosomal storage diseases

Xi Zoë Zhong[1], Xue Sun[1], Qi Cao[1], Gaofeng Dong[1], Raphael Schiffmann[2], and Xian-Ping Dong[1, *]

SUPPLEMENTARY FIGURES

Figure S1. Activation of BK reduces lipofuscin in NPC1 cells in a dose/time -dependent manner. (A) Activation of BK with NS1619 (16 hrs) inhibited abnormal accumulation of lipofuscin (detected by autofluorescence) in NPC1 human fibroblasts in a dose-dependent manner. (B) Activation of BK with NS1619 (15 μM) inhibited abnormal accumulation of lipofuscin in NPC1 human fibroblasts in a time-dependent manner. (C) Activation of BK with NS11021 (16 hrs) inhibited abnormal accumulation of lipofuscin in NPC1 human fibroblasts in a dose-dependent manner. More than 40 cells were analyzed for each condition.

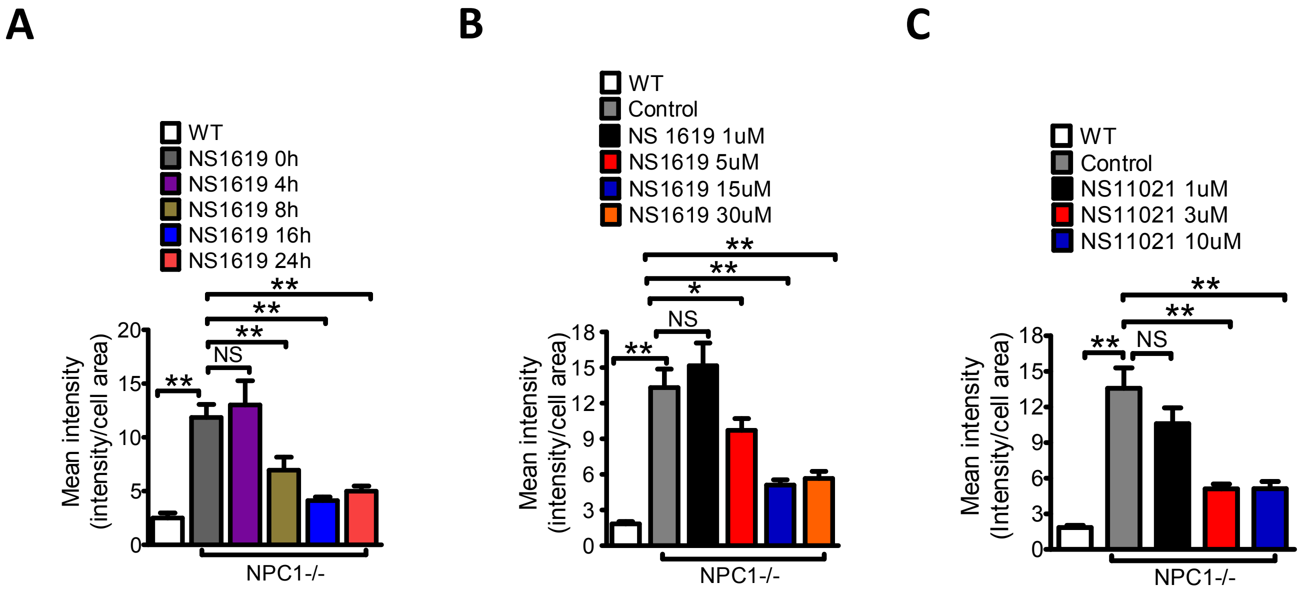
A
WT
NS1619 0h
NS1619 4h
NS1619 8h
NS1619 16h
NS1619 24h
Mean intensity
(intensity/cell area)
20
15
10
5
0
**
NS
NPC1-/-
B
WT
Control
NS 1619 1uM
NS1619 5uM
NS1619 15uM
NS1619 30uM
Mean intensity
(intensity/cell area)
18
15
12
9
6
3
0
**
*
NS
NPC1-/-
C
WT
Control
NS11021 1uM
NS11021 3uM
NS11021 10uM
Mean intensity
(Intensity/cell area)
18
15
12
9
6
3
0
**
NS
NPC1-/-

Figure S1
